# An isolated *Mycobacterium tuberculosis* vulvar lesion: A case report

**DOI:** 10.1016/j.imj.2025.100217

**Published:** 2025-11-16

**Authors:** Caren Challita, Nour El Moussawi, Maya Dagher, Nelly Rubeiz, Souha S. Kanj

**Affiliations:** a Division of Infectious Diseases, Department of Internal Medicine, American University of Beirut Faculty of Medicine, Beirut 11072020, Lebanon; b Department of Dermatology, American University of Beirut Faculty of Medicine, Beirut 11072020, Lebanon; c Center for Infectious Diseases Research, American University of Beirut, Beirut 11072020, Lebanon

**Keywords:** Vulvar tuberculosis, Extrapulmonary tuberculosis, Genital tuberculosis, *Mycobacterium tuberculosis*, Vulvar lesion

## Abstract

Vulvar tuberculosis is an exceptionally rare manifestation of extrapulmonary *Mycobacterium tuberculosis* infection. We describe a 28-year-old woman from Republic of the Philippines presenting with a painful vulvar lesion of 3 months' duration unresponsive to several courses of antibiotics for a presumed sexually transmitted disease. A diagnosis of isolated vulvar *Mycobacterial tuberculosis* was made based on findings of caseating granulomas on vulvar biopsy and positive mycobacterial culture. The patient achieved complete resolution following standard antituberculous therapy. This case highlights the importance of suspecting tuberculosis in chronic vulvar lesions from endemic areas and underscores tissue biopsy and prolonged culture when initial stains are negative.

## Highlights

• Uncommon presentation of tuberculosis as an isolated persistent vulvar ulcer.
• Diagnostic delay due to mimicry with sexually transmitted diseases.
• Successful treatment with standard anti-tuberculosis therapy.
• Importance of considering extrapulmonary TB in unusual genital lesions.

## Graphical abstract

**Figure:**
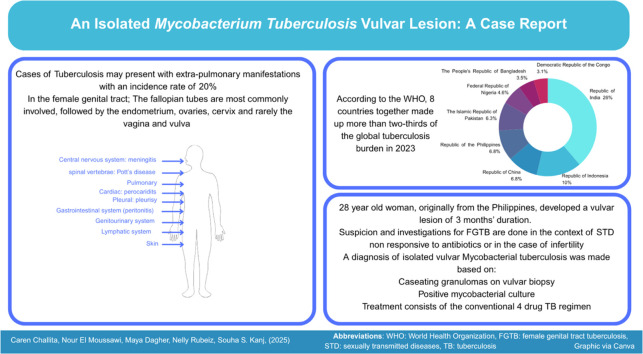

## 1 Introduction

While being a preventable and curable disease, tuberculosis (TB) is currently the deadliest disease worldwide from a single infectious organism. In fact, in 2023, as reported by the World Health Organization, 8.2 million newly diagnosed cases were registered, reaching a new record since 1995, with 1.25 million deaths recorded.^1^ Commonly known as a pulmonary disease, cases may present with extra-pulmonary manifestations with an incidence rate of 20%, and may involve any organ system including the female genital tract.^2^ Women at risk of developing female genital tract tuberculosis often come from endemic countries, mainly low to middle income countries, or have a history of TB exposure. Other risk factors include an immunocompromised state, malnutrition, intravenous drug use or human immunodeficiency virus (HIV) infection.^3^^,^^4^ Individuals may present with constitutional symptoms alongside organ-specific manifestations, mimicking other infectious processes, making diagnosis more challenging.^2^^,^^5^ We describe a patient who had an indolent vulvar lesion for a course of 3 months, secondary to *Mycobacterium tuberculosis,* highlighting its atypical presentation.

## 2 Case report

A 28-year-old Filipino woman, previously healthy, who immigrated to the Lebanese Republic (Lebanon) 10 years ago as a domestic worker presented to the infectious diseases' clinic with a 3 months' history of a painful single vulvar lesion, associated with a burning sensation especially upon micturition. No associated respiratory or gastrointestinal symptoms and no fever were reported. The patient denied any sexual activity within 6 months preceding the appearance of the lesion.

Upon her employment, a purified protein derivative (PPD) test showed induration > 10 mm with a clear chest X-ray, consistent with latent TB infection in the context of a high-burden country (the Philippines). She declined latent TB treatment after being counseled on the risks of reactivation and was advised to follow up regularly with her physician for monitoring. She had already received a course of amoxicillin-clavulanic acid prescribed empirically by a healthcare provider at another facility for a presumed skin and soft tissue infection in the vulvar area. No bacterial cultures were obtained at that time. She had also used topical antibiotics, and steroid creams with no improvement.

**Fig. 1:**
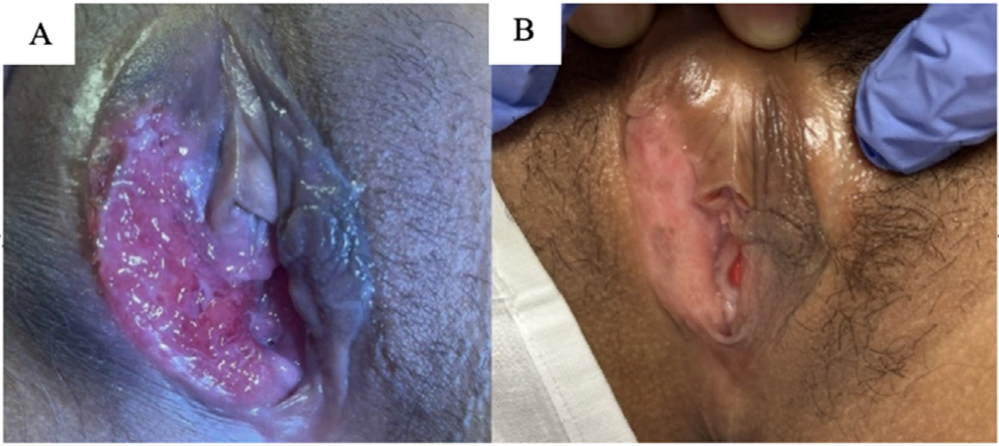
Vulvar lesion. (A) Vulvar lesion upon presentation. (B) Vulvar lesion after 2 months of combination treatment.

Physical examination revealed a tender, erythematous, vegetative, indurated and ulcerated plaque on the right vulvar area eroding the labia minora (Fig. 1A). A tender right inguinal lymph node was palpable.

A polymerase chain reaction (PCR) panel for sexually transmitted diseases (STD) was taken. The panel tested for *Neisseria gonorrhoeae, Chlamydia trachomatis, Mycoplasma genitalium, Mycoplasma hominis, Ureaplasma urealyticum, Ureaplasma parvum, Trichomonas vaginalis*, herpes simplex virus types 1 and 2 (HSV-1, HSV-2), *Treponema pallidum*, and *Gardnerella vaginalis*. Meanwhile, a 21-day course of doxycycline was empirically initiated by the dermatology department to treat the possibility of chancroid as the diagnosis is often clinical. At the end of the course, she reported symptomatic improvement, but the lesion persisted.

HIV serology and Venereal Disease Research Laboratory (VDRL) test came back negative, and the STD panel was positive for only *Gardnerella vaginalis*. She was prescribed a seven-day course of metronidazole.

With the lack of improvement, she was referred to a gynecologist for a Papanicolaou test smear and biopsy. Three vulvar punch biopsies were taken for regular bacterial cultures and pathology. Cultures grew *Enterococcus feacalis* and *Streptococcus anginosus*. Vulvar biopsy revealed non-caseating granulomas and negative acid-fast bacilli (AFB), Grocott's methenamine silver, and periodic acid-Schiff stains. No changes suggestive of malignancy were seen.

The patient was referred to a dermatologist for further evaluation. Stool calprotectin ordered for the suspicion of cutaneous Crohn's disease was mildly elevated. A magnetic resonance imaging of the pelvis showed significant subcutaneous inflammation in the vulva with no intestinal pathologies or fistula. In addition, a right inguinal adenopathy measuring 12 mm was appreciated, along with a subserosal pedunculated uterine fibroma measuring 11.4 mm. The patient was evaluated by a gastroenterologist specializing in inflammatory bowel diseases, including Crohn's disease. After a comprehensive clinical assessment and a detailed review of the patient's medical records, Crohn's disease was considered highly unlikely in this case, and no further work-up was pursued.

**Fig. 2:**
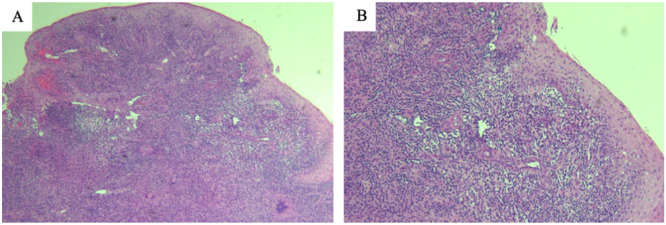
Histopathological examination of the specimen taken from the vulva. (A) Hematoxylin and eosin stain showing diffuse granulomatous inflammatory cell infiltrate (4× magnification). (B) Hematoxylin and eosin stain showing diffuse granulomatous inflammatory cell infiltrate (20× magnification).

A second punch biopsy was performed for mycobacterial and fungal. Hematoxylin and eosin stain (Fig. 2) showed epidermal parakeratosis, scale-crust formation, spongiosis, exocytosis of neutrophils and irregular hyperplasia. The dermis had a diffuse granulomatous inflammatory cell infiltrate composed of epithelioid cells, multinucleated giant cells of the foreign body and Langhans types with an admixture of neutrophils, nuclear debris and mononuclear cells distributed within and around these granulomas. The findings were suggestive of granulomatous diseases, most likely granulomatous tuberculoid dermatitis. AFB staining was performed on this sample and was negative for AFB. Gram, PAS and Giemsa stains were non-revealing. Additional investigations, including PCR for *M. tuberculosis* and routine laboratory markers such as complete blood count, C-reactive protein, and erythrocyte sedimentation rate, were not performed due to financial constraints. Thirty-six days after incubation, the vulvar biopsy culture grew *M. tuberculosis*, which was identified using standard laboratory methods at our laboratory which uses the BACTEC Mycobacterial Growth Indicator Tube (MGIT) system for TB detection, liquid media Middlebrook 7H9. The patient was started on isoniazid, rifampin, pyrazinamide, and ethambutol with pyridoxine.

Two months later, she returned for follow-up and the lesion showed complete epithelialization (Fig. 1B). Ethambutol and pyrazinamide were discontinued, as the isolate was susceptible to isoniazid and rifampin, which were continued for four additional months.

## 3 Discussion

Female genital tract tuberculosis is an uncommon extra-pulmonary manifestation of *M. tuberculosis*. It is suspected in the context of STDs unresponsive to multiple courses of antibiotics or infertility.^6^

The fallopian tubes are most commonly involved (90%–100%), followed by the endometrium (50%–80%), ovaries (20%–30%), cervix (5%–15%) and rarely the vagina and vulva (1%–2%).^7^ Although it mostly affects women of reproductive age, postmenopausal cases have also been reported, endorsing the potential role of hormones in the disease development. However, vulvar TB can occur at any age (from 7 months to 85 years).^6^

Infection may spread by direct inoculation (e.g., sexual intercourse), hematogenous or lymphatic spread from primary involved organs (lungs, lymph nodes or bowels) with a latency period ranging from 5 to 40 years.^3^^,^^8^ In our patient, reactivation of untreated latent tuberculosis with hematogenous or lymphatic spread to the vulva appears most plausible, given her prior positive PPD and absence of recent sexual activity.

Vulvar TB may present as a non-healing ulcerative and hypertrophic lesion, with or without suppuration and ulceration of the inguinal lymph nodes.^3^^,^^9^ Differential diagnosis may include elephantiasis vulva, severe mycoses, lymphogranuloma venereum, chancroid, syphilis, herpes simplex virus, leishmaniasis, Lipschütz ulcer and malignancy.^9^^,^^10^ In our case, the patient's transient relief after doxycycline contributed to the initial diagnostic challenge. This temporary improvement may be attributed to doxycycline's partial anti-inflammatory effect and its antibacterial activity against concomitant colonizing organisms, as earlier cultures had grown *Enterococcus faecalis* and *Streptococcus anginosus*. However, the persistence of the lesion despite treatment highlighted the need to pursue alternative diagnoses, ultimately leading to the recognition of TB.

To date, there are no clear guidelines for the diagnosis of vulvar TB. A high level of suspicion is key especially in endemic areas. Detailed history, physical examination and adequate laboratory work-up should be performed.^7^ Confirmation of extrapulmonary TB requires a positive culture, positive histopathology or clinical evidence of active disease.^11^ AFB smears require at least 10,000 organisms/mL in the sample and have limited use in a paucibacillary disease. Hence, cultures are more sensitive, requiring 10–100 mycobacteria/mL but necessitate around 2–8 weeks of incubation on Lowestein-Jensen medium. This explains why AFB staining remained negative in our patient despite repeated attempts, as genital TB is typically paucibacillary. The eventual growth of *M. tuberculosis* on culture highlights the importance of persisting with culture (and PCR when available) in cases with strong clinical suspicion. On the other hand, PCR testing is highly sensitive and enables detection of rifampin resistance. Unfortunately, PCR testing was not performed in our patient due to financial and accessibility constraints. Finally, histopathology may show epithelioid cells and multinucleated giant cells surrounding a caseating granuloma, which in the right clinical setting, can be highly suggestive of TB.^12^^,^^13^

The patient's untreated latent TB infection, documented by a prior positive PPD, raises the likelihood that this case represents reactivation. This highlights the importance of completing latent TB therapy in individuals from endemic regions, even in the absence of immunosuppression, to prevent reactivation and unusual extrapulmonary manifestations.

Treatment consists of the four-drug regimen (rifampin, isoniazid, pyrazinamide, and ethambutol) for the first 2 months followed by a combination of rifampin and isoniazid for 4 months tailored to susceptibility. The response to treatment can be seen by regression and resolution of the lesions, but documentation of treatment success with histopathological examination is preferred in the case of residual lesions.^8^

## 4 Conclusion

An isolated vulvar lesion should raise the suspicion for extra-pulmonary TB, especially in patients from endemic regions, even in the absence of previous signs or symptoms of TB infection. Prompt antibiotic therapy is essential while assessing potential multi-drug resistance.

## CRediT authorship contribution statement

**Caren Challita:** Writing – original draft, Investigation, Conceptualization. **Nour El Moussawi:** Writing – original draft, Investigation, Data curation. **Maya Dagher:** Writing – original draft, Visualization, Project administration. **Nelly Rubeiz:** Writing – review & editing, Validation, Investigation. **Souha S. Kanj:** Writing – review & editing, Supervision, Conceptualization.

## Informed consent

Informed consent was obtained from the patient for the publication of the case details and accompanying images. All efforts were made to ensure patient anonymity, and no identifiable personal information has been disclosed.

## Organ donation

Not applicable.

## Ethical statement

This case report was reviewed by the Institutional Review Board (IRB) of the American University of Beirut, which determined that it does not meet the definition of human subject research and therefore did not require IRB approval.

## Data availability statement

Data sharing is not relevant to this article as no datasets were generated or analyzed during the study.

## Animal treatment

Not applicable.

## Generative AI

Generative artificial intelligence tools were used solely for grammar and spelling checks. All content was written by the authors and subsequently reviewed to ensure accuracy and integrity.

## Funding

This research did not receive any specific grant from funding agencies in the public, commercial, or not-for-profit sectors.

## Declaration of competing interest

The authors declare no conflict of interest.
